# Digital healthcare services in the context of age-friendly community initiatives in China: a realist evaluation

**DOI:** 10.1186/s12913-025-13846-8

**Published:** 2025-12-08

**Authors:** Yefei Zhao, Guanglin Lu, Shuai Fang, Lingying Cai, Xiaolin He, Yan Liang

**Affiliations:** 1 School of Nursing, Fudan University, Shanghai, 200032 China; 2 Institute of Sociology, Shanghai Academy of Social Sciences, Shanghai, 200020 China; 3 Department of Social Policy, Shanghai Administration Institute, Shanghai, 200233 China

**Keywords:** Digital care, Person-centered care, Qualitative research, Older adults, Home and community-based

## Abstract

**Background:**

Understanding digital healthcare services in age-friendly community (AFC) initiatives and their implementation is crucial for developing evidence-based policies and practices. In this study, we aim to explore the question, “What is it about digital healthcare services that work, for whom, and under what circumstances?” in top-down AFC initiatives using Shanghai, China, as a representative case.

**Methods:**

We employed a novel realist evaluation methodology and gathered insights from critical informants and users, including policymakers, community officers responsible for implementing AFC initiatives, and older adults.

**Results:**

The findings revealed six context-mechanism-outcome configurations. The government-led top-down AFC initiative, along with its related policies, serves as a critical context for supporting the provision of digital healthcare services and improving outcomes for older adults and their communities. A bottom-up approach values individual demand-oriented methods, providing digital healthcare services in AFC initiatives by considering older adults’ health and social situation as a critical context, utilizing digital care services and support as resources, and enhancing user experience. A feasible path forward is to support healthcare professional-led collaboration in developing and implementing digital healthcare services, aligning bottom-up and top-down practices, and focusing on individual demand, social equity, privacy concerns, and data-driven feedback.

**Conclusions:**

Top-up support, active engagement of providers, and closer local monitoring of digital healthcare service implementation in AFC initiatives may encourage long-term effectiveness and sustainability.

**Supplementary Information:**

The online version contains supplementary material available at 10.1186/s12913-025-13846-8.

## Background

The rapid growth of the older population presents significant social and healthcare policy challenges, including the need for living situations that accommodate mobility impairments, the provision of supportive services, and the facilitation of social connections that increase with age [1]. In response, aging has become a dominant policy driver, focusing on supporting older adults to live better in their homes and communities [2]. Age-friendly community (AFC) initiatives have been launched worldwide over the past decade [3] to promote the well-being of older residents and improve the quality of life of the entire community [4].

Digital services have rapidly developed in recent years to address global healthcare and social welfare issues [5]. They are attracting considerable interest in AFC initiatives because of their potential as effective and cost-efficient options for care services and support for older individuals [6–8]. However, adopting digital technologies poses considerable challenges for older adults, which have been largely overlooked [9]. As individuals age, their cognitive abilities gradually decline, making it increasingly difficult to adapt to the rapid development of digital technology [10]. Seifert et al. suggest that older adults are less likely to utilize telemedicine opportunities because of a lack of internet usage or relevant skills [11]. Additionally, Chong and Chiu reported that older adults who lack connections with family and friends via the Internet experience more loneliness, less satisfaction, and severe mental health problems [12]. The digital divide among older populations [13] necessitates a deeper understanding of digital healthcare services from the perspective of older adults in AFC initiatives.

Recent studies on digital services in AFCs have primarily focused on the application of digital technology. For example, the “Smart Age-friendly Ecosystem” is a blueprint for aging-friendly environments [14], the “Mobility Digital Ecosystem” is a viable solution to enhance urban transportation for older individuals [6], and the “PlaceCal” project establishes community information networks to promote social participation among older persons [15]. Other studies have focused on interventions to improve the inclusion of digital environments and promote equity and justice by proposing appropriate solutions and recommendations to address these issues [16]. These studies provide practical foundations. However, few researchers have discussed the implementation process and the mechanisms by which digital healthcare services in AFCs lead to change.

Understanding digital healthcare services within the context of AFC initiatives and their implementation is crucial for developing evidence-based policies and practices. China implemented AFC initiatives in 2020, aiming to have 5,000 demonstrations of urban and rural AFCs nationwide by 2025 and achieve full coverage by 2035 [17]. The Committee on Aging of the China National Health Commission is responsible for these initiatives. A four-stage plan was created: preliminary demonstration (2020–2022), follow-up demonstration (2023–2025), summary stage (2026–2030), and evaluation stage (2031–2035) [17]. Top-down AFC initiatives focus on six aspects: living environment, daily travel, quality of services, social participation, spiritual and cultural life, and technology-supported services (e.g., intelligent care, telemedicine, and digital health services) [17]. Digital transformation has been highlighted in China’s 14th Five-Year Plan (2021–2025), aiming to stimulate data-driven dynamics and embark on a new journey toward a digital China [18]. Thus, the case of China provides an excellent opportunity to understand digital healthcare services in the AFC initiatives. This will facilitate the development of digital services in China and provide insights into improving these services in other countries with similar top-down older adult care programs.

Top-down and bottom-up approaches are classical models in policy implementation literature, and many studies [19–22] successfully applied these models to understand China’s policy implementation. For example, by integrating top-down state regulatory pressure and bottom-up worker-enterprise interaction model, Chung [19] reveals the dual root causes of contemporary China’s labor law compliance dilemma. There are structural faults in the government enforcement mechanism, and a strategic avoidance behavior based on survival rationality exists in basic labor relations subjects, which constitutes the continuous tension between the legal text and its practical implementation. Van Rooij [20] reveals the formation mechanism of the “weak law-enforcement and strong compliance” paradox in China’s tax field by integrating the top-down institutional deterrence mechanism and the bottom-up social constraint effect. Gallagher [21] examines top-down and bottom-up perspectives on how Chinese law protects labor rights while maintaining political control and promoting strong economic growth. Qian et al. [22] investigate the status of social insurance coverage of informal sector workers in a bottom-up manner to test the importance of business compliance in promoting the expansion of social insurance coverage. This offers valuable insights into China’s AFC initiatives from a public policy perspective. However, both top-down and bottom-up approaches have drawbacks as intervention mechanisms. While the top-down approach is better at providing coordination, it may lead to disengagement and demotivation of government actors, insufficient response to local challenges, and low levels of user support. While a bottom-up approach can stimulate the participation of social actors, it would reduce economies of scale by creating a more specific regulatory framework. Especially in healthcare, we still face the challenge that once implementation is achieved in one setting, it becomes increasingly difficult to replicate elsewhere [23]. From the perspective of implementation science in healthcare [24], it is critical to understand how to get evidence into practice. Existing efforts to understand the implementation process and thereby facilitate the generalization of implementation strategy success have relied on dividing and studying contextual factors, theoretical mechanisms, and outcomes in isolation. Realist research paradigms provide an analytical tool for articulating the explicit role of context within the causal process, moving beyond a catalog of preconditions for implementation success [23]. This study employs a realistic evaluation to explore the synergy between top-down and bottom-up approaches and their impact on the implementation of digital healthcare services in AFCs from the context-mechanism-outcome interaction perspective.

This study examines how realist evaluation can illuminate the implementation processes and mechanisms through which digital healthcare services in AFCs bring about change. Rooted in theory-driven [25] inquiry, realist evaluation seeks to answer questions such as “What works, for whom, under what conditions, and how?” within healthcare research [26]. The methodology identifies assumptions or existing theories about how specific program components might produce outcomes. These theories then shape research questions and data collection strategies. Data is analyzed through iterative exploration of context-mechanism-outcome configurations, refining initial theories to explain what works, for whom, why, and under which circumstances. The result is a nuanced theory that integrates empirical evidence and interpretations [27], emphasizing explanation over mere description, aligned with realist scientific principles [28]. Table 1 outlines key realist concepts and this study’s alignment with realist philosophy. In healthcare interventions, the top-down approach typically involves policy-driven strategies, while bottom-up approaches center on clinician-led initiatives [29]. These are occasionally framed as distinct pathways in literature, reflecting differing levels of implementation—systemic versus frontline practices. Top-down and bottom-up approaches are sometimes alternatively used as top-down and bottom-up pathways or processes in healthcare intervention literature [30–32].

**Table 1:** Definitions of terms

Terms	Definition
Realist methodology	It is a method grounded in theory and interpretation that seeks to reveal the foundational logic or principles shaping interventions and their components, while examining how contextual conditions interact with change mechanisms to generate specific outcomes.
Initial program theories	Realist evaluations examine and refine theories that explain how programs achieve their goals. All initiatives are based on underlying assumptions—whether stated or unstated—about how their components lead to desired outcomes. While initial program theories may not align with realist principles, the evaluation process adapts them into realist program theories by clarifying how specific contexts activate mechanisms that drive outcomes. This involves iteratively testing and revising the theory to account for the dynamic relationships between context, mechanism, and impact.
Context-mechanism-outcome configurations (CMOCs)	CMO configuring is an iterative, experience-driven method that identifies causal explanations for outcomes observed in data. These configurations can apply to an entire program or focus on specific elements. CMOs may overlap, with one nested within another, or they might be structured sequentially. Constructing CMOs serves as a foundation for developing or refining the theoretical framework that ultimately shapes the conclusions of the analysis.
Top-down approach	A “top-down” approach involves centralized decision-makers, such as government authorities or policy specialists, formulating and disseminating proposals to stakeholders. This method prioritizes strategic policy planning by offering detailed guidelines, well-defined roles for implementation, and streamlined processes with minimal bureaucratic hurdles. A government-driven, top-down strategy is particularly effective for enhancing the core systems of AFC infrastructure, strengthening its foundational requirements, developing digital frameworks, and achieving large-scale adoption of digital solutions—all while accelerating project timelines.
Bottom-up approach	A “bottom-up” approach begins by defining policy objectives and reverse-engineering the roles of stakeholders and institutional frameworks needed to drive behavioral change. Instead of relying on strict hierarchical control, this method assumes that effective implementation hinges on policymakers identifying which parts of an organizational ecosystem are best suited for specific tasks, what resources are required to execute them, and how these actions influence the behaviors of the target population. Community-focused, bottom-up strategies are advantageous for aligning AFC initiatives with local needs and priorities. They foster high levels of public engagement with lower resistance to execution, while also enhancing communities’ capacity for self-management and decision-making.

AFC age-friendly community

This study proposes an initial program theory (Table 2) that elucidates digital healthcare services in AFC initiatives based on previous literature (Supplemental Table S1), creative thinking sessions among the research team, and stakeholder consultation. The initial program theory is based on the World Health Organization’s Integrated People-Centred Health Services framework (WHO’s IPCHS framework) [33], and we also referred to the digital entrepreneurial ecosystem framework [34], highlighting the importance of user involvement. As AFC initiatives [35, 36] and digital services [5] face sustainability challenges, particularly the implementation gap between early development stages and long-term viability, it is crucial to involve older adults in structuring digital healthcare systems in a more person-centered, demand-driven manner. The initial program theory (highlighting the underlying mechanisms that provide the essential elements to facilitate change) empowers and engages people and communities, strengthens governance and accountability, reorients the model of care, coordinates services within and across sectors, and creates an enabling environment [33]. This will facilitate integrated, people-centered digital healthcare services in the context of AFC initiatives in China.

**Table 2:** Mechanisms

Initial program theory mechanism	Refined program theory mechanism	The response prompted by digital healthcare services in China’s AFC initiatives	Influence pathways
Empowering and engaging people and communities	Individual demand-oriented	Individual demand-oriented digital care services and support benefit the user experience.	Individual demand-oriented
	Privacy concerns	Incorporating privacy considerations in AFC infrastructure development can improve the user experience.	Individual demand-oriented
Strengthening governance and accountability	Social equity considerations	Considerations of social equity They are an essential factor in the governmental allocation of digitalized AFC infrastructure and ultimately affect the user experience of vulnerable populations.	Government-led
Reorienting the model of care	Digital care services and support	Individual demand-oriented services, collaboration, and data-driven feedback during realist implementation foster digital care services and support, influencing user experience, resource integration, and efficiency improvement.	Individual demand-oriented, government-led, and professional-led
	Data-driven feedback	Data-driven feedback improves efficiency.	Needs individual demand-oriented and professional-led
Coordinating services within and across sectors	Collaboration	Digital care services, support, and the AFC infrastructure improve resource integration and efficiency via collaboration.	Professional-led
Creating an enabling environment for healthcare services	AFC infrastructure	AFC infrastructure can benefit user experience and resource integration via privacy and social equity considerations and collaborative implementation processes.	Government-led

AFC age-friendly community

## Aim

To explore “What is it about digital healthcare services that work, for whom, and in what circumstances” in top-down AFC initiatives using China as a representative case and the perspectives of key informants and users (policymakers, community officers, and older adults).

## Methods

We used a realist evaluation methodology. As a theory-driven approach, realist evaluation transcends exploring the effectiveness of an intervention [25]. We chose Shanghai to examine the mechanisms of digital healthcare services under China’s AFC initiatives. Shanghai was one of the first pilot AFC cases in China. The efforts to promote AFCs are guided by central government guidelines, which outline the basic principles, set development goals, clarify key tasks, and outline support measures. The program theory defines the scope of the evaluation (Table 2). The study design was based on the principles of realist evaluation [25, 37] and guided by RAMESES II reporting standards for realist evaluations [28]. This study was approved by the Research Ethics Committee of Fudan University, School of Nursing (IRB#2023-4-7). Written informed consent was obtained from all participants before the interview.

Data were collected between September 2023 and December 2023. The methods used included one-on-one in-person interviews and documentary reviews. Each interview took 40–70 min, depending on the information the interviewee wished to share, and was conducted in Mandarin. All interviews were audio recorded. Interviewers took field notes and wrote memos during or after the interviews. Through these interviews, older adults were asked to reflect on their experiences with and expectations of digital healthcare services through AFC initiatives. Additionally, district and community officers were invited to consider the local expertise in digital healthcare services within the AFC initiatives (see the semi-structured interview topic guide in online supplementary file 1, Table S2). The reviewed documents included policy documents and community-level materials, with the following details: policy documents covered municipal and district-level regulations on AFC or digital services, including those related to remote healthcare, in-home hospital services, and internet-plus nursing; community-level materials included work reports on AFC and typical case records. To ensure the comprehensiveness of document sources, we first invited district and community officials to provide relevant documentation. Additionally, the research team independently searched for and collected relevant materials through various channels.

To understand how digital healthcare services in the context of China’s AFC initiatives are implemented, purposive sampling was used. Roles and districts guided the purposeful sampling, and three groups were approached for interviews, representing users and sources of critical information. The inclusion criteria for participants were as follows:

1. Older adults involved in digital healthcare services in AFCs.
2. District officers, including the chief of AFC management from four districts in Shanghai (Changning District, Pudong New District, Putuo District, and Xuhui District), as their role as policymakers comprises planning and guiding AFC implementation.
3. Community officers, as they were responsible for implementing digital services in particular areas of AFCs.
4. The ability to communicate, give consent, and participate.

Exclusion criteria included individuals with serious diseases who may not complete the entire interview.

The interview data were digitally recorded, transcribed, and anonymized. All the data were imported into NVivo software for management. Thematic analysis was employed for data analysis [38]. We used inductive and deductive coding, based on theories, and open coding for the new themes identified, seeking information to explain the contexts, mechanisms, and outcomes [28, 39]. Coding was completed independently by two authors (YZ and GL), and regular meetings with the research team were held to debate and challenge the interpretations of the evidence and propose alternative explanations (see Example of themes and sub-themes in online supplementary file 1, Table S3).

## Results

We conducted 20 interviews with older users (12), district AFC management (4), and subdistrict management (4). (see the participants’ general information in online supplementary file 1, Table S3 and Table S4)

**Fig. 1:**
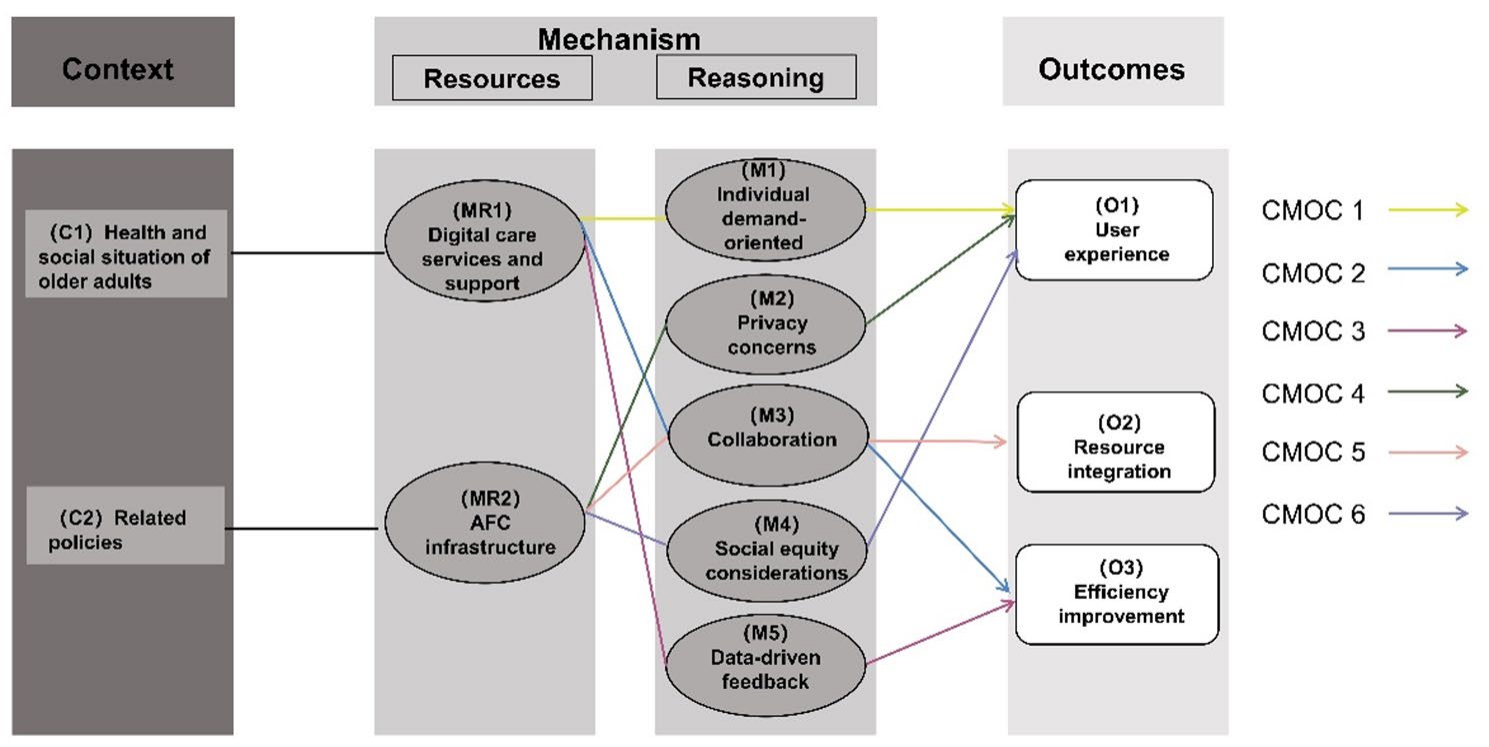
Interrelated context-mechanism-outcome configurations (CMOCs) for how digital healthcare services in age-friendly community (AFC) initiatives work from the perspectives of key informants and users

The findings are organized into two sections: (1) the observed outcomes, including user experience, resource integration, and efficiency improvement, and (2) the grouped context-mechanism-outcome configurations (CMOCs), showing how the mechanisms explain the outcomes. These findings were grouped into six CMOCs (Fig. 1).

### Observed outcomes from the perspectives of key informants and users

#### User experience

Key informants and users recognized user experience as an essential outcome of digital health services. District officers described user experience as a buffer for older adults’ hesitancy concerning digital health service use.

Some older people are reluctant to use digital technology, but if they have a good user experience, or are surrounded by relatives or friends who have a good experience with it, they will want to try it out…. (D1)

Other user experiences mentioned by key informants and older adults included reduced loneliness, increased social engagement, aging in place, access to healthcare, and feeling safe at home.

“Tmall Genie has many other functions. If the elderly want to listen to Shanghai TV dramas and dialogues, it can take on such a role and give them spiritual comfort.” (C5) “I can use the ‘One Touch Call’ service to call a car when I go out, and if I fall or something, I can also call for emergency help, which is very convenient and makes me feel safe….” (O8) “In a nursing home, I am not free to go out….at home, I can make an appointment with a community doctor online, which makes me feel more free (independent) than living in a nursing home.” (O12).

#### Resource integration

Resource integration was also identified as an outcome of digital health services. The community integrates and optimizes the public infrastructure resources to provide services for older adults. This facilitates the medical treatment and travel needs of older adults in both online and physical environments. By optimizing the online medical treatment and drug dispensing process, remote digital treatment platforms and physical and mental health consultation services are provided to balance the proportion of online and offline medical resources.

Health or medical insurance resources, grassroots empowerment of resource integration… these aspects allow people to be one stop away from enjoying the convenience of physical space. (C6)

#### Efficiency improvement

District and community officers viewed efficiency improvements as a key outcome of digital health services. Through digital support facilities, data sharing facilitates the daily management of community staff. Sharing data enhances information transparency and acceptability. The remote digital treatment platforms and alarm facilities save human resources, meet medical treatment needs, and improve the efficiency of community services.

The city’s system serves as a source of recording statistical data, and the community can use the data to facilitate daily management and improve management efficiency. (D3)

Telemedicine is a kind of intuitive face-to-face technology that helps you solve problems, save personal energy, and eliminate journeys. (C8)

### CMOCs

#### Developing individual demand-oriented digital care services and support

CMOC 1: Individual demand-oriented digital care services and support benefit the user experience.

The initiative’s context was characterized by the involvement of stakeholders in the health and social situation of older adults (context). This prompted district and community officers to develop individual, demand-oriented digital care services and support to address the challenges faced by older adults (mechanism). Consequently, individual demand-oriented digital care services benefit the user experience (outcome).

Resources: Digital care services and support. To meet the social and medical care needs of community-dwelling older adults, a series of digital healthcare services and support was provided. These services and support were viewed as supplementary resources rather than necessities. Additionally, digital-related training was provided for older adults who were willing to adopt a digital lifestyle but required more relevant skills. The combination of digital and traditional care services, along with the introduction of digital technology, has increased the accessibility and convenience of community healthcare.

“I can book a home visit from my family doctor online, which is convenient.” (O5). “Community health center will call me regularly to check my self-management of hypertension.” (O1). “We have community elder care consultants who will introduce and explain our digital healthcare services and policies to the elders…We don’t ask older adults to adapt to digitalization; if they are willing to, we provide the appropriate resources, and if they don’t want to, we still keep the traditional services.” (C4) “We provide training, such as smart cellphone use, for the elders. The training is designed and organized according to the characteristics of the older population.” (C3)

Mechanism: Individual demand-oriented services. This approach is critical when implementing digital healthcare in AFC initiatives. It influences the design and delivery of the service, relating them to the experiences of older adults.

“Older adults in the community have a common demand for getting prescription medications regularly from their family doctors. We set up smart medicine cabinets in the community to interface with the services of family doctors.” (C3) “To meet the communication needs of older adults, a Terminal Large Screen was built in the neighborhood stops to help older residents who can’t use a smartphone to call a taxi or make an appointment with doctors, and older adults can one-push-call on the Terminal Large Screen to access the community call center to get assistance.” (C2) “I can give feedback to our community on my phone based on my needs and problems.” (O3) “I prefer living at home rather than in elder care institutions if community services can meet my needs.” (O12)

#### Developing collaboration in digital care services and support

CMOC 2: Collaboration in digital care services and support improves efficiency.

The initiative’s context was characterized by stakeholders involved in the health and social situation of older adults (context). This prompted providers to collaborate in addressing the challenges faced by older adults (mechanism). Consequently, professional-led collaboration benefits efficiency improvement (outcome).

Mechanism: Collaboration. Collaboration was viewed positively, and the interviewees identified the challenges and benefits of efficiency improvement.

We used these smart devices to help early warning; however, the major challenge in practice is a collaborative follow-up service rather than device use themselves…We are considering the possibility of collaboration among healthcare providers, community workers, and the third-party community property management companies to benefit the collaborative response. (D2)

#### Increasing data-driven feedback in digital care services and support

CMOC 3: Data-driven feedback in digital care services and support for improved efficiency.

The initiative’s context was characterized by stakeholders involved in the health and social situation of older adults (context). This prompted community members to increase data-driven feedback on digital care services and provide support to address the challenges that older people were facing (mechanism). Consequently, data-driven feedback benefits efficiency improvement (outcome).

Mechanisms: Data-driven feedback. The consensus from district and community officers was that data-driven feedback can improve efficiency.

I think the data may benefit statistics analysis and decision making on the municipal level, while facilitating daily work management on a community level, to improve efficiency. (D3)

#### Rising privacy considerations in the AFC infrastructure

CMOC 4: Privacy considerations in the AFC infrastructure benefit the user experience.

The initiative’s context was characterized by stakeholders involved in related policies (context). Government-led top-down policy implementation is beneficial for optimizing the AFC infrastructure. Moreover, privacy concerns in the AFC infrastructure (mechanism) benefit the user experience (outcome).

Resources: AFC infrastructure. During the interviews, it was evident that government-led digital services in AFC initiatives broadly promoted digital infrastructure, which interviewees described as utilized resources. This example demonstrates that the digital infrastructure in AFC initiatives encompasses smart community dining, telemedicine, monitoring devices to prevent disorientation, and emergency response at home.

Installation of Internet of Things (IoT) sensors, fall warning and alarm devices, and smoke alarms for the seniors living alone who are willing to (D2). When a community is retrofitted with smoke alarms, other communities spread the word by visiting the demonstration, which is conducive to subsequent scale-up. (C4)

Mechanism: Privacy concerns. Community officers believe privacy considerations in the AFC infrastructure influence user acceptance of digital services.

“Smart meter monitoring will set an alarm threshold based on the amount of water used over some time, and the alarm will be triggered when too much or too little is used, but the acceptance of some residents is not as high as expected, and residents will have privacy concerns.” (C4) “For wearable devices, such as bracelets that monitor vital signs, some older adults or their families are willing to use them; some are not because of privacy concerns.” (C2)

#### Developing collaboration in the AFC infrastructure

CMOC 5: Collaboration in the AFC infrastructure benefits resource integration.

The initiative’s context was characterized by stakeholders involved in related policies (context). Government-led top-down policy implementation is beneficial for optimizing the AFC infrastructure, and collaboration in this infrastructure (mechanism) benefits resource integration (outcome).

Mechanism: Resource integration. District and community officers believed that collaboration within the AFC infrastructure benefits resource integration and that government-led models alone are insufficient.

“We are doing a collaborative project–– Internet Plus Hospital–– in our community elder care center this year to integrate social care and medical care.” (D1) “It will not be sustainable if we only depend on the government.” (C2)

#### Rising social equity considerations in AFC infrastructure

CMOC 6: Social equity considerations in AFC infrastructure benefit user experience.

The initiative’s context was characterized by stakeholders involved in related policies (context). Government-led top-down policy implementation is beneficial for optimizing the AFC infrastructure, and social equity considerations within the AFC infrastructure (mechanism) ultimately benefit the user experience (outcome).

Mechanism: Social equity considerations. District officers viewed the most critical component for the older population as social equity considerations, which will benefit users, especially vulnerable older people.

We carried out a project–smart care for living alone–to ensure safety for that population, including one-push-call, at-home three-alarm toolkits (infrared warning system, smart water meter warning, and smoke alarms). The target population should be prioritized as the vulnerable older people, who may expand to other needy populations. (D2)

### Summary of the CMOCs

**Fig. 2:**
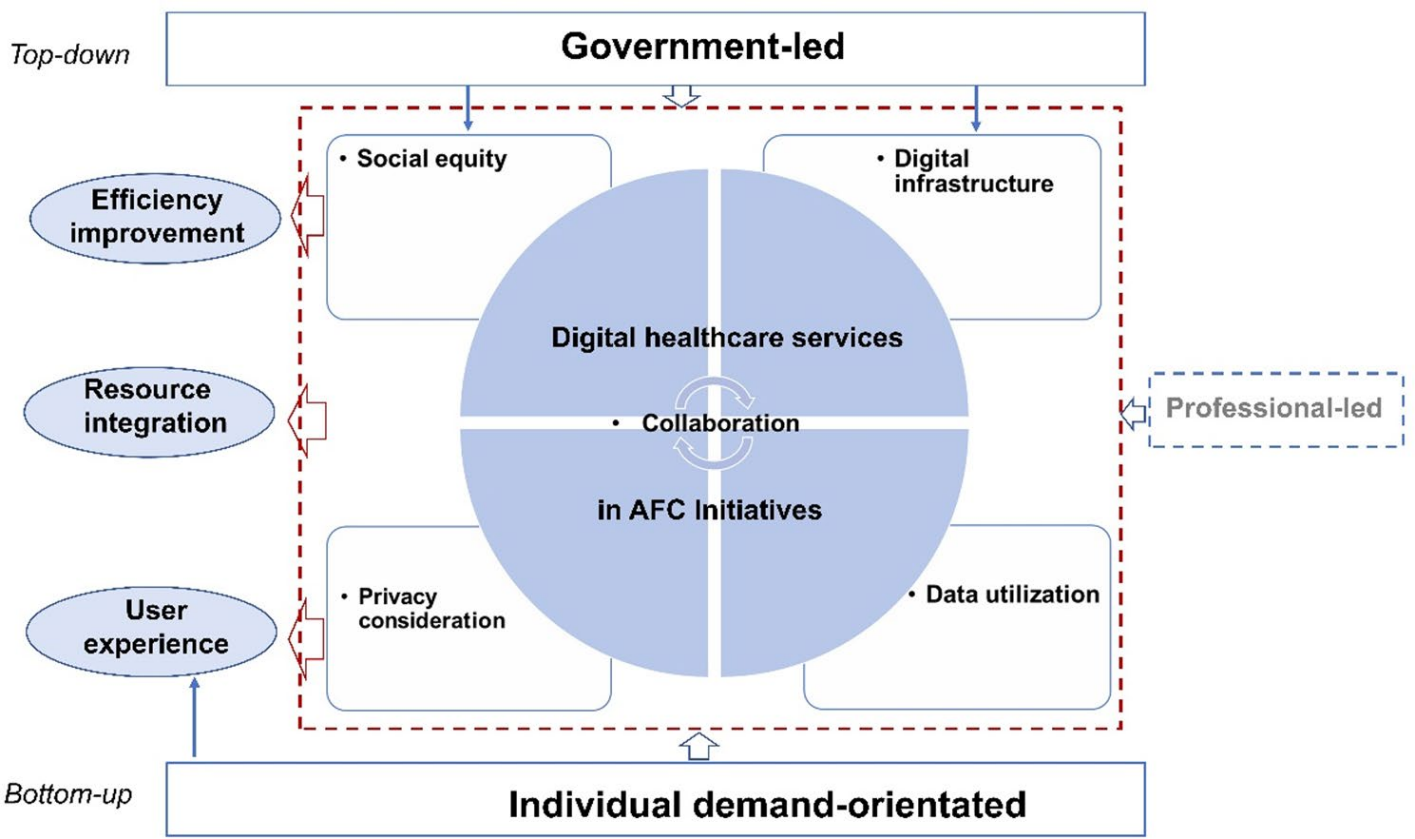
A simplified model of digital healthcare services in age-friendly community (AFC) initiatives

**Fig. 3:**
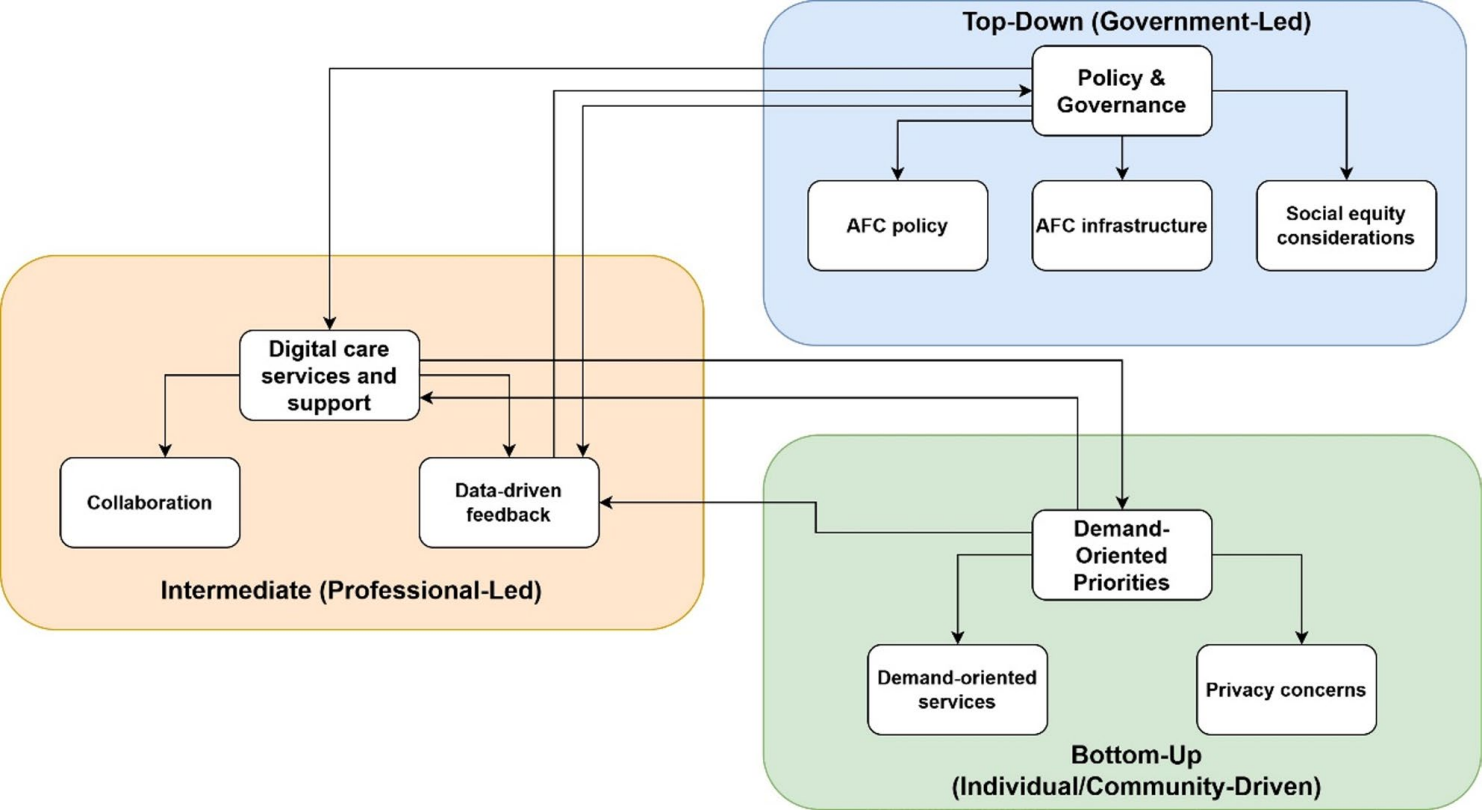
An illustration of the interplay between top-down and bottom-up approaches

Based on the identified CMOCs, we propose a simplified model of digital healthcare services in AFC initiatives (Fig. 2) to illustrate the core elements and appropriate course of action. We also illustrate key findings, including the interplay between top-down and bottom-up approaches, in Fig. 3.

Government-led, top-down AFC initiatives and related policies are crucial for supporting digital healthcare services and enhancing outcomes for older adults and their communities. This prompted district policymakers to regard the AFC infrastructure as a significant resource that values social equity to improve user experience. In addition, as part of the AFC infrastructure, privacy concerns and collaboration issues emerged as essential challenges in implementing digital services, as these influenced outcomes, including user experience, resource integration, and efficiency improvement. Second, the bottom-up approach prioritized individual demand-oriented methods of providing digital healthcare services in AFC initiatives, valuing older adults’ health and social situation as a critical context, digital care services and support as resources, and improving user experience. Collaboration and data-driven feedback were also valued for enhancing user experience outcomes, resource integration, and efficiency improvement. Collaboration exists in both mechanisms, indicating an essential bridging role in providing digital healthcare services in AFC initiatives, which requires further exploration in future studies.

## Discussion

The study explored “What is it about digital healthcare services that work, for whom, and in what circumstances” in top-down AFC initiatives. Building upon the WHO’s IPCHS framework [33], we focus on the five components of the theory (Initial program theory mechanism in Table 2) and further develop the WHO’s IPCHS framework [33] by unpacking the context and mechanisms of digital healthcare services in AFC initiatives (Refined program theory mechanism in Table 2). Figure 1 summarizes six CMO statements that connect them to the findings of digital healthcare services in AFC initiatives, specifically regarding context, mechanisms, and outcomes.

Our empirical data (Fig. 1) shows that the top-down AFC approach influences the effectiveness of digital healthcare services in the context of related policies, with AFC infrastructure as resources, reasoning through privacy concerns, collaboration, and social equity considerations; the bottom-up approach works in the context of health and social situation of older adults, with digital care services and support as resources, reasoning through individual demand-oriented, collaboration, and data-driven feedback. Digital care services, support, and AFC infrastructure serve as resources and influence the implementation of integrated, people-centered digital healthcare services in the context of AFC initiatives in China.

The views of users and key informants support the program theory that elucidates digital healthcare services in the context of the AFC initiatives in China. Collaboration may be a pivotal mechanism linking top-down and bottom-up approaches to facilitate digital healthcare delivery in top-down AFC initiatives. This study is the first to identify the core elements of digital healthcare services in top-down AFC initiatives and their potential mechanisms. Notably, the proposed elements are not mutually dependent. Commitment to collaboration and careful consideration of individual demand-oriented approaches, social equity, privacy concerns, and data-driven feedback may positively impact digital healthcare service delivery in AFC initiatives.

Our findings support individual demand-oriented approaches and social equity as critical mechanisms for explaining what works in digital healthcare services in AFC initiatives. Previous studies [40, 41] have highlighted the importance of considering the needs of older adults when introducing digital technology. Our analysis revealed that an individual demand-oriented approach significantly influenced user experience, particularly when considering the specific health and social situations of older adults. The digital service needs of older adults should be identified and incorporated into the design process of the digital environment [42]. Moreover, healthcare professionals should understand the perspectives and motivations of older adults regarding the use of digital services. This knowledge can significantly facilitate older adults’ eventual acceptance of digital services [43]. Promoting social equity is another critical mechanism of digital services and support, particularly from a top-down perspective. The characteristics of older adults (e.g., age, sex, and socioeconomic status) can impact their access to digital healthcare services [44]. It is essential to provide targeted and accessible digital services for subpopulations to narrow social inequalities in health services [45]. However, research has shown that digital services lacking clear targets may exacerbate disparities [44]. Therefore, involving vulnerable older adults in the co-creation of digital services and focusing on addressing existing inequalities can promote social inclusion and reduce injustice. Overall, to bridge the gap in digital healthcare access, a multifaceted approach is essential. This includes implementing targeted subsidies for low-income populations [46], establishing mobile health units to serve rural communities, and launching culturally sensitive outreach programs. Equally critical is the adoption of inclusive design principles in digital healthcare platforms, which guarantees that these services are accessible and user-friendly for vulnerable populations [47], thereby promoting equitable healthcare delivery.

Collaboration has been highlighted as a critical mechanism for digital healthcare services in AFC initiatives. This informs the potential “bridge” role in linking top-down and bottom-up approaches. AFC initiatives involve several stakeholders, including end users (older adults, caregivers, and other residents), supervisors (representatives from government and policy-making institutions), and those in the construction sector (real estate developers and project investors) [48]. Digital healthcare services also involve community, clinical, and hospital professionals to deliver collaborative and tailored health services for older persons [49]. By setting up multi-stakeholder task forces, creating shared platforms for communication [50], and formulating joint accountability frameworks [51], collaboration between stakeholders can be effectively enabled and maintained—thus helping to resolve challenges such as conflicting priorities and resource allocation issues. Such collaboration can allow older individuals to continue caring for themselves and delay their admission to nursing homes owing to their declining physical abilities and ailments [52]. Healthcare professionals have the potential to actively collaborate to promote the two previously discussed mechanisms in digital healthcare services in AFC initiatives. For example, collaborating with older adults and their family members to improve their digital health literacy [53], engaging in community advocacy and policy initiatives to bridge the gap between current development stages and long-term sustainability [36], and facilitating multidisciplinary co-creation during healthcare service development can ensure the successful implementation and sustainability of digital services [5]. Actionable long-term sustainability strategies include funding models (e.g., public-private partnerships [54, 55], subscription-based models [56]), policy adjustments (e.g., incentives for digital adoption [57], regulatory frameworks [58]), and scalability considerations (e.g., infrastructure development [59], technology integration [60]).

Our study also identified the roles of privacy concerns and data-driven feedback in digital healthcare services. Previous research has reported privacy concerns among older adults regarding remote monitoring [61], which has resulted in diminished trust in digital healthcare interactions [62]. Despite this apprehension, older individuals have confidence in sharing their health data with trusted healthcare professionals and providers [63]. To minimize the negative impact of privacy concerns on digital healthcare service use, several measures may be helpful, such as equipping older adults with better knowledge of information and communication technologies [64], considering older adults’ privacy concerns when designing digital services, customizing the activities and applications they request, and implementing personal data protection measures to reduce data privacy breaches and increase their acceptance of digital support services [61, 65, 66]. Specific recommendations include implementing robust data encryption [67], adopting anonymization techniques [68], establishing clear ethical guidelines for data usage [69], and launching user education campaigns to raise awareness about data privacy rights [70].

Data-driven feedback was considered when designing and implementing digital care services and support, which promotes efficiency gains. Data analysis and feedback can identify variations in healthy aging, provide tools to track changes that support age-friendly efforts in the region [71], and enhance services, support, and inform policy modifications, leading to the continuity and effectiveness of changes over time. Data-driven feedback plays a pivotal role in enhancing digital healthcare services by systematically analyzing end-user usage data to identify service gaps, employing advanced analytics to optimize resource allocation, and integrating user feedback into iterative design processes. For instance, specific health needs, such as unique drug request functionalities, client-led viral load calculators, remote consultation requests, and drug delivery features, can be identified through data analysis within healthcare applications [72]. Additionally, user engagement can be bolstered by incorporating feedback mechanisms, such as rating systems and motivational symbols (e.g., stars, checkmarks), which have been shown to improve adherence and user satisfaction. From a policy perspective, the implementation of Data-Driven Innovation (DDI)—a structured seven-step framework encompassing product conceptualization, data acquisition, data refinement, data storage and retrieval, distribution, presentation, and market feedback [73]—provides a robust foundation for developing analytics-driven service delivery models that are both efficient and scalable.

The refined program theory mechanism enables change with differing impacts. Different aspects of digital healthcare services in AFC have sparked various mechanisms in other settings, with varying effects on older adults and the community. The evaluation has shown that elements of multifaceted digital healthcare services in AFC can work independently, and that the local context (including local challenges faced) should shape how digital healthcare services in AFC are implemented. This study reinforces the point that successfully implementing digital healthcare services in the AFC requires combining the introduction of AFC infrastructure and digital care services with adequate support and management mechanisms. It is also important to highlight that professionals play a significant role in the success of the intervention, as individual demand orientation, privacy considerations, and data utilization are crucial dimensions. This finding aligns with those of other studies [74–76]. The findings will help replicate implementation successes from one healthcare setting to another.

### Strengths and limitations

This is the first study to employ realist methods in focusing specifically on digital healthcare services within top-down AFC initiatives. Its strength is that it reflects the reality of policy and practice to facilitate the understanding of how, why, when, and what makes digital healthcare services in AFC initiatives “complex.”

This study had several limitations that should be considered. First, our representative case was limited to Shanghai, rather than the rest of China. This may limit the generalizability of our findings. Despite the limitations of case selection, this realist evaluation, as a theory-driven methodology, was built on theories developed from the existing evidence base, and the proposed program theory was tested and refined using empirical data. This can improve the transferability and usefulness of the research findings beyond the local context. Still, we encourage future research to expand the geographic scope by including data from other cities or regions, such as tier-2 cities or rural areas, where digital healthcare adoption may differ significantly. This would enhance the external validity of the findings and provide a more comprehensive understanding of digital healthcare services in China. Second, the evaluation scope of this study was primarily focused on the perspectives of key informants, including policy and practice experts (district and community officers) and users (older adults), and did not include frontline healthcare professionals. Studies are needed to explore healthcare professionals’ perspectives further, understand collaboration mechanisms, and investigate the policy implementation gap. Incorporating interviews or focus groups with healthcare professionals in future research would offer a more holistic view of the digital healthcare ecosystem. This would help identify barriers to implementation, training needs, and strategies for improving service delivery, ultimately enriching the study’s findings. Third, the limitations of the realist evaluation methodology. While this approach offers valuable insights into the “what works, for whom, and under what circumstances” framework, it is not without its challenges. Realist evaluation heavily relies on qualitative data, which, while rich in context, may lack the generalizability and measurable impact that quantitative data provides. This limitation restricts the ability to draw broadly applicable conclusions or statistically validate findings. Isolating mechanisms within complex social systems, such as digital healthcare services in AFC initiatives, can be challenging. The interplay of multiple factors—contextual, individual, and systemic—makes it difficult to definitively attribute outcomes to specific mechanisms. We acknowledge these limitations and recommend complementing realist evaluation with mixed-methods approaches in future research. This would enhance the robustness of findings by combining qualitative depth with quantitative breadth.

While this study is geographically limited to Shanghai, its realist evaluation framework identifies transferable insights applicable to other contexts. The six context-mechanism-outcome configurations offer a robust foundation for implementing digital healthcare services in age-friendly community (AFC) initiatives, particularly in regions with similar top-down governance and community-based healthcare models. Key findings, such as the importance of tailoring services to older adults’ health and social contexts, fostering healthcare professional-led collaboration, and integrating data-driven feedback, are universally adaptable. To enhance applicability, we recommend conducting pilot studies across diverse regions, engaging local stakeholders, and collaborating internationally to refine strategies and share best practices.

### Actionable policy recommendations

Based on our findings, we propose the following strategies to improve the implementation of digital healthcare services in AFC initiatives: Foster partnerships among policymakers, healthcare professionals, and community organizations to align top-down and bottom-up approaches; for example, establish regular forums for dialogue and feedback. Develop context-specific digital healthcare solutions by conducting needs assessments in diverse regions, including rural and tier-2 cities, to address varying demographic and infrastructural challenges. Provide targeted training for healthcare professionals and older adults to improve digital literacy and ensure effective use of digital healthcare tools. Implement feedback mechanisms to collect usage data and patient outcomes, enabling continuous service improvement and addressing privacy concerns through robust data protection measures. Design inclusive policies that prioritize accessibility for marginalized groups, such as low-income older adults, by subsidizing digital devices and internet access. Establish monitoring frameworks to track the implementation and impact of digital healthcare services, ensuring accountability and long-term sustainability.

## Conclusions

Improving digital healthcare services in AFC initiatives remains a top priority. This realist evaluation identifies the critical mechanisms that explain what works in digital healthcare services within AFC initiatives. Supporting healthcare professional-led collaboration in the development and implementation of digital healthcare services, aligning bottom-up and top-down practices, and focusing on individual demand, social equity, privacy concerns, and data-driven feedback can be a feasible approach. Top-up support, active engagement of providers, and closer local monitoring of digital healthcare service implementation in AFC initiatives may encourage long-term effectiveness and sustainability.

## Supplementary Information

Below is the link to the electronic supplementary material.

Supplementary Material 1

## Data Availability

The datasets generated during and analyzed during the current study are not publicly available, as respondents were assured that the raw data would remain confidential and not be shared.
