# Supplementary material for: Digital healthcare services in the context of age-friendly community initiatives in China: a realist evaluation
Source: BMC Health Serv Res. 2025 Dec 8;26:58. doi: 10.1186/s12913-025-13846-8 (PMC12797359; doi:10.1186/s12913-025-13846-8)
Supplement: Supplementary file 1 — Supplementary Material 1 [file 12913_2025_13846_MOESM1_ESM.docx]

**Digital healthcare services in the contexts of China’s Age-friendly Communities Initiatives: a realist evaluation**

Online supplemental 1

Table S1. The literature-derived potential programme theories

Table S2. The semi-structured interview topic guide

Table S3. Example of themes and sub-themes

Table S4. The participants’ general information- older adults

Table S5. The participants’ general information- district and community officers

Table S1. The literature-derived potential programme theories

| Programme theories | Literature | Fileds |
| --- | --- | --- |
| - COM-B framework, designed for Behaviour change, describes the three domains of: Capability: physical skills, knowledge, behavioural regulation and memory, attention and decision process; Opportunity: environmental context, resources and social influences; Motivation: beliefs about consequences, optimism and beliefs about capabilities and reinforcement and emotion. - RFV framework, designed for the successful implementation of telecommunications technology health interventions, describes the three domains of: Relationships: with health workers and peer as a means of providing support for behavioural change, feedback and reinforcement; Fit: integration of mHealth into routine and its ease of use into the existing environment and Visibility: to engage in information to mediate and motivate self-management tasks and enable enhanced awareness. | Watkins JA, Goudge J, Gómez-Olivé FX, Huxley C, Dodd K, Griffiths F. mHealth text and voice communication for monitoring people with chronic diseases in low-resource settings: a realist review. Bmj Glob Health. 2018;3(2). | Digital services |
| - Differentiation between consequences for the actor(s) and others is mediated through the culture and social structure. This conceptual review discusses consequences that i) disrupt power relations between patients and health professionals or between different groups of health professions, ii) contribute to paradoxical outcomes, and iii) result in a potentially corrosive subculture of pessimism about digital health. | Ziebland S, Hyde E, Powell J. Power, paradox and pessimism: On the unintended consequences of digital health technologies in primary care. Social Science & Medicine. 2021;289. | Digital services |
| - The theoretical framework of governance structure, was proposed in the third level of sociological research classification, and compared to a spectrum. The left end of the spectrum is the market governance structure, the right end is the government (hierarchy) governance structure, and the middle is the hybrid governance structure (HGS). The governance model will be based on a bottom-up style when approaching the left end. On the contrary, when getting closer to the right end, the governance model is led by the government in a top-down style with a low level of public participation, in which the public is more passively informed about rather than actively involved. | Liu JS, Zhang XF, Lin J, Li YR. Beyond government-led or community-based: Exploring the governance structure and operating models for reconstructing China?s hollowed villages. Journal of Rural Studies. 2022;93:273-86. | Top-down initiatives |
| - The WHO- IPCHS framework proposes five interdependent strategies that need to be adopted for health service delivery to become more integrated and people-centred. The five interconnected strategies are: (1) Engaging and empowering people and communities. (2) Strengthening governance and accountability. (3) Reorienting the model of care (i.e., ensuring that efficient and effective healthcare services are designed, purchased, and provided through innovative models of care). (4) Coordinating services within and across sectors. (5) Creating an enabling environment (i.e., in order for the four previous strategies to become an operational reality, it is necessary to create an enabling environment that brings together all stakeholders to undertake transformational change) | Ahmed A, van den Muijsenbergh METC, Mewes JC, Wodchis WP, Vrijhoef HJM. Untangling the inter-relatedness within integrated care programmes for community-dwelling frail older people: a rapid realist review. Bmj Open. 2021;11(4). | Community-based elder care services |
| - Normalisation process theory (NPT), is a theory of implementation that focuses on the work people do surrounding the implementation of new sets of practices. NPT proposes four constructs, ‘generative mechanisms’, which characterise different types of work that ‘people do as they work around a set of practices’. The four NPT constructs comprise: coherence ‘sense- making work’, cognitive participation ‘relational work to build and sustain a community of practice’, collective action ‘operational work to enact a set of practices’ and reflexive monitoring ‘formal and informal assessment of the new sets of practice’. | Alharbi K, Blakeman T, van Marwijk H, Reeves D, Tsang JY. Understanding the implementation of interventions to improve the management of frailty in primary care: a rapid realist review. Bmj Open. 2022;12(6). | Community-based elder care services |
| - Organisational Readiness Theory, defines organisational readiness as a multilevel and multifaceted construct referring to organisational members’ shared commitment to change—encompassing both willingness and capacity. This readiness for change is crucial in producing collective engagement; that is achieving buy in and commitment from those at the front lines enacting the change. This engagement results in valuable implementation outcomes: a collective commitment to initiate change, greater effort to make the change successful, greater perseverance when barriers are encountered and an increase in pro- social collaborative behaviours that promote the change. | Long JC, Sarkies MN, Auton EF, Nguyen HM, Pomare C, Hardwick R, et al. Conceptualising contexts, mechanisms and outcomes for implementing large-scale, multisite hospital improvement initiatives: a realist synthesis. Bmj Open. 2022;12(5). | Large-scale initiatives |
| - Bronfenbrenner’s ‘socio-ecological systems’ framework, sought to conceptualize how individuals are both affected by, but also affect, their environment at different nested levels from the nano- and micro- to the exo- and macrosystems. The nano-system comprises the genetics and personal characteristics of the individual, while the microsystem includes the immediate environment in which they interact. The mesosystem connects these structures, and the exosystem encompasses agencies within the wider social system that affect opportunities for social and civic participation. The macrosystem comprises societal elements that shape culture, values, policies, and laws, and the chronosystem accounts for transitions within the system, especially within the temporal dimension. Together, these levels offer a comprehensive understanding of the systems that influence individuals in their personal and social contexts | Sixsmith J, Makita M, Menezes D, Cranwell M, Chau I, Smith M, et al. Enhancing Community Participation through Age-Friendly Ecosystems: A Rapid Realist Review. Geriatrics-Basel. 2023;8(3). | Age-Friendly initiatives |

Table S2. The semi-structured interview topic guide

| **Participants** | **Question** |
| --- | --- |
| Older adults | 1、Recall your use of digital health services   Your health and social situation (briefly describe your daily life; For example, how are daily life activities, social activities, what social roles are undertaken, etc., and the use of technical support)   What event or problem caused the need for care or use of digital health services (triggers, acute accidents such as falls or illnesses)? Can you tell us more about the situation at that time?   What changes have occurred in the arrangements at home after the need for someone to take care of them (family care, hiring part-time workers, etc.)? What assistive technologies are used? How is housekeeping assistance solved? What practical help and support have been received (what formal care services have been used, such as home community elder care services/long-term care insurance services/Internet plus care, hospital at home, etc.)? How do you use digital-supported medical care (chronic illness, medication, treatment, etc.)? Personal care (bathing, eating, etc.) |
|  | 2、In the age-friendly community, your perceptions, feelings, and experience of using digital health services   What is the most significant change for you or your family before and after use? Can you tell me specifically? (from the following aspects: personal feelings; daily life; physical activity; changes in time spent on some daily activities; changes in social participation or social roles)   Your opinion on these digital technologies themselves (reliability, lifespan, functionality, purchase and maintenance costs). What do you think about the reliability of these technologies? How long do you think it will last? What about the features? What do you think about the purchase price and later maintenance?   Your opinion on the effects of using the technology (the impact on you, the impact on your family or caregiver). What are the effects on you after use? What are the effects (daily activities, self-care, etc.) on the family? Was it helpful? The good or the bad aspects can be discussed.   Your self-efficacy (ability to use; expected effect). Did you have any difficulties in using the technology? Do you think you will use it? Or can you learn it? What kind of results do you expect to achieve?   Concerns during use |
|  | 3. What other needs have you and your family in terms of care (including social care and medical care) that have not been met? Please talk about it specifically. |
| District officers | 1. Please tell us about the policy background and specific projects of the age-friendly communities in your district, especially digital health services. |
|  | 2. What are the specific considerations for the design and implementation of projects in the district? For example, target population, operation mechanism, management mechanism, etc.? |
|  | 3. What are the challenges and problems encountered in the implementation of projects and services? Please give an example. |
|  | 4. What results have been achieved by the project and services? What are some areas that need further improvement or refinement? |
| Community officers | 1. Please tell us about the specific implementation of the construction of age-friendly communities and digital health services in this street. |
|  | 2. What resources are provided in the implementation of projects and services? |
|  | 3. What are the specific practices in service integration, information integration, and multi-party collaboration? The result? |
|  | 4. What are the specific problems and challenges encountered? What are some suggestions for improvement and refinement? |

Table S3 Example of themes and sub-themes

| Themes | Sub-themes | Citation |
| --- | --- | --- |
| Context | Health and social situation of older adults  Current status of digital management  Policy background  Current status of AFC | "The community hospital is really good. They often come to our homes to check on our medication intake, reminding us not to forget to take our hypertension medicine. They also frequently call to ask about our blood pressure." (O1)  "We can make online appointments for home-based community services and sign up with a community family doctor. But the family doctor in charge of our residential area has too many people to look after—they’re swamped." (O5)  "This year, another integrated medical and elderly care program was added, promoting the 'Internet + Hospital' model. It’s part of the comprehensive elderly service center." (P1)  "We have added sensing devices, namely IoT (Internet of Things) sensing devices, and made minor adjustments to the traditional care and response processes. The focus is on the safety of elderly people living alone, precise assistance, fall early warning and alarm for the elderly, and prevention of elderly wandering. For the elderly living alone care program, smart mattresses and smoke sensors are used to trigger emergency rescue." (P2)  "There are both certain compatibility and contradictions between digitalization and the elderly. In terms of age structure, it is obvious that the older the elderly, the generally lower their acceptance of digitalization." (C8)  "There are fall prevention alarms (installed by the neighborhood committee) and one-click call devices (which can send messages to the neighborhood committee and the elderly’s children). Now there are also external one-click call devices; these are used for seeking help from outside." (O3)  "The three-year action plan for the 'Smart Highland Strategy' defines the goals of smart elderly care as: using intelligence to assist the elderly, enable the elderly, and honor the elderly. For the digital transformation of elderly care, Changning District’s Smart Elderly Care Information Platform was officially launched in 2019." (P4)  "The 'Digital Partner Program' involves entities with network infrastructure that can provide digital technology services. With the upgrading and transformation of phone booths, the digital large screens (installed there) support one-click ride-hailing—elderly people can easily get a taxi as long as they are at these stations." (C6)  "Elderly care consultants present information on digitalization-related policies." (C8)  "Every day, each doctor checks in on and cares about the elderly’s condition. If there’s an issue, they will come (in person); if not, they provide regular telemedicine services. As for the AI canteen, it uses robots for cooking—applying digital technology and artificial intelligence to standardize Chinese cuisine in elderly care services. This is a quite distinctive feature." (C6)  "Medication dispensing is a top priority, as there is a large demand for it. There are smart medicine cabinets that connect to family doctor services." (C7)  "We hope someone can come to the home to assist the two elderly people with daily tasks, such as meal preparation, bathing and dressing, and household cleaning." (O1) |
| Mechanism | Individual demand-oriented  Supportive resources  Privacy concerns  Social equity considerations  Supporting services and Public-private cooperation  Data-driven feedback | "When it comes to medication dispensing and taking, I have no one to assist or remind me. I go to the hospital for regular check-ups, but it’s not very convenient since my health is poor. For emergencies, I usually call an ambulance." (O1)  "The 'family call' function means elderly people don’t need to remember their children’s phone numbers—this is because, as we mentioned earlier, some elderly people may have disabilities. Smart home devices are used to convey information to the elderly." (C6)  "We should consider the elderly’s proactive needs; for example, the additional features mentioned could be incorporated into age-friendly renovations, including smart elderly care initiatives. The main goal of these efforts is to ensure the elderly’s safety." (C7)  "Under the elderly smart technology enhancement program, we provide on-site guidance to the elderly on using smart devices to increase their adoption rate. Additionally, we have optimized micro-tourism routes through elderly-friendly smart technology." (P5)  "We offer smart technology support to help the elderly learn to use smartphones, including specialized training on smartphone operation. However, the requirements and methods for teaching the elderly should not be the same as those for children or students—they must adapt to the elderly’s specific habits and behaviors." (C7)  "We have produced many training videos and provided them to the elderly, so they can watch whenever they need to review. Since their children often lack the time, patience, and energy to teach them digital functions, we offer guidance and services, as well as deliver policy-related information." (C8)  "Elderly people have a low level of adoption of digital health monitoring devices. They either don’t know how to use them, or are unwilling to use them due to a subjective resistance to being monitored." (P4)  "For elderly activity trackers, some elderly people are willing to use them, or their family members are willing to urge them to use them. However, elderly people generally have a certain degree of resistance to such wearable smart devices." (C6)  "Smart water meters, for example, will trigger an alarm if water consumption is abnormally high or low for an extended period. But residents’ acceptance of this device is not high, as they feel it invades their privacy." (C8)  "This year, we have added an 'integrated medical and elderly care' program to the comprehensive elderly service centers and promoted the 'Internet + Hospital' model." (P1)  "To adapt to digitalization, we provide corresponding resources for those elderly who are willing to embrace it; for those who are not, we also retain services that meet their non-digital needs to fill the gap." (C8)  "After we installed smoke detectors in one community, other communities learned from it by visiting this demonstration community. This has effectively fostered a positive atmosphere for such initiatives." (C8)  "The service process is transparent: information is sent to guardians to subject elderly care institutions to social supervision. Dining data can be monitored; once rules are triggered, an incident alert is activated. The meal assistance management system is included in online supervision, and food traceability is implemented to improve elderly people’s satisfaction." (P3)  "There is a digital hotline circle that enables one-click access to hotline services. The 114 service now offers appointments for renowned doctors and handles non-emergency transportation requests, with direct human operators available. Smart home devices not only convey information to the elderly but also allow them to call for help externally." (C6)  "The one-click outbound call service is specifically for elderly people living alone—they can report any issues directly via their mobile phones." (O3)  "First, we need to break monopolies and promote free competition. The municipal government can also give platforms the opportunity to participate, but I believe these platforms should compete fairly—no specific platform should be designated." (P1)  "It remains unclear who should be the final responsible party between the neighborhood committee secretary and the assigned manager: once terminal services are involved, who should take charge, and to what extent? Additionally, there is the issue of the property management company’s response. A legal relationship is established after installations are completed, so it is worth considering who should take responsibility if no one responds." (P2)  "When it comes to elderly care services, relying solely on the government to provide a safety net is unsustainable." (C6)  "Dining data can be monitored; once predefined rules are triggered, an incident alert will be activated immediately." (P3)  "Traditional alarms have a lag: for example, if an elderly person has an accident, it may take 2 to 3 days to detect it. Currently, such incidents can be detected within 24 hours. Smart water meters analyze water consumption against preset thresholds through a backend system and send alert messages to guardians promptly." (P4)  "For alarm monitoring devices, there is currently a challenge: some elderly people do not use smartphones, making it impossible to record data." (C8) |
| Outcomes | User experience  Resource integration  Efficiency improvement | "Devices like Tmall Genie have many other functions. If elderly people want to listen to Shanghai opera or have a conversation, it can serve this role and provide them with emotional comfort." (C5)  "Elderly people can use smart devices to avoid feeling lonely. The community also regularly organizes activities to honor and respect the elderly. With these safeguards in place, I believe the safety level for elderly people living at home and going out has improved." (C7)  "In nursing homes, elderly people can’t go out freely. On one hand, the staff won’t allow it; on the other hand, their children are unwilling to pick them up. Being cooped up inside all the time naturally makes them feel unhappy. It’s still more comfortable to receive elderly care at home." (O12)  "For health and medical insurance resources, we need to integrate resources along the line of grassroots empowerment, starting with the integration of physical spaces. This way, residents can enjoy the convenience of one-stop services in a single physical location." (C6)  "The online medication dispensing mechanism needs to be further improved. Since we are promoting online medication dispensing, the regulations should be more detailed, and each step of the online operation process should be well-designed." (C7)  "Digital support services can compensate for many missing functions in terms of quality of life. Building such a community (with digital services) benefits us as service providers, and brings even greater benefits to the elderly." (C8)  "The municipal-level system serves as a platform for data statistics and recording, while communities can use this data to facilitate daily management and improve management efficiency." (P3)  "Meal assistance management system: It is integrated into online supervision and food traceability systems to enhance elderly people’s satisfaction. It also provides monitoring coverage, data sharing, and cross-verification to support unified management and sharing of data." (P3)  "Telemedicine is not the intuitive in-person interaction; instead, it solves issues through face-to-face communication via a screen. Firstly, it saves individuals’ energy, and secondly, it reduces the hassle of traveling to medical facilities." (C8) |

Table S4. The participants' general information- older adults

| **Number** | **Age-ranges (years)** | **Sex** | **Education Level** | **Marital Status** | **Income**  **(CNY/Month)** |
| --- | --- | --- | --- | --- | --- |
| 1 | 75-84 | Female | High School | Divorced | 5001-10000 |
| 2 | 75-84 | Female | College | Married | 3001-5000 |
| 3 | 75-84 | Male | College | Married | 5001-10000 |
| 4 | 75-84 | Male | College | Married | 3001-5000 |
| 5 | 75-84 | Female | College | Married | 5001-10000 |
| 6 | 75-84 | Female | Postgraduate | Married | >10000 |
| 7 | 85+ | Female | Junior School | Married | 3001-5000 |
| 8 | 75-84 | Female | Junior School | Married | >10000 |
| 9 | 85+ | Female | College | Widowed | 5001-10000 |
| 10 | 85+ | Female | Junior School | Widowed | 5001-10000 |
| 11 | 85+ | Female | Undergraduate | Widowed | 5001-10000 |
| 12 | 85+ | Male | Junior School | Married | >10000 |

Table S5. The participants' general information- District and community officers

| **Number** | **Age-ranges (years)** | **Sex** | **Education Level** | **Role** | **Area** |
| --- | --- | --- | --- | --- | --- |
| 1 | 40-50 | Male | Postgraduate | District officer | Pudong |
| 2 | 40-50 | Female | Postgraduate | District officer | Xuhui |
| 3 | 40-50 | Male | Postgraduate | Community officer | Xuhui |
| 4 | 40-50 | Female | Postgraduate | District officer | Changning |
| 5 | 40-50 | Male | Undergraduate | District officer | Putuo |
| 6 | 40-50 | Male | Postgraduate | Community officer | Changning |
| 7 | 40-50 | Female | Undergraduate | Community officer | Changning |
| 8 | 40-50 | Male | Undergraduate | Community officer | Changning |
